# Supplementary material for: Identification of photoperiod-induced specific miRNAs in the adrenal glands of Sunite sheep (Ovis aries)
Source: Front Vet Sci. 2022 Jul 22;9:888207. doi: 10.3389/fvets.2022.888207 (PMC9354845; doi:10.3389/fvets.2022.888207)
Supplement: Supplementary file 1 [file Data_Sheet_1.ZIP › Supplementary file/miRDeep2 file.zip!/miRDeep2 file/Know_Structure/SP42A1/oar-mir-29b-1.pdf]

miRBase precursor : oar-mir-29b-1
Total read count : 7632
oar-miR-29b read count : 7010
remaining reads : 622

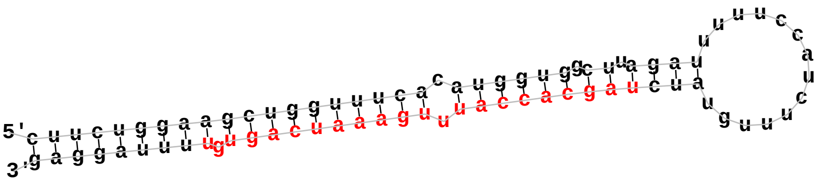

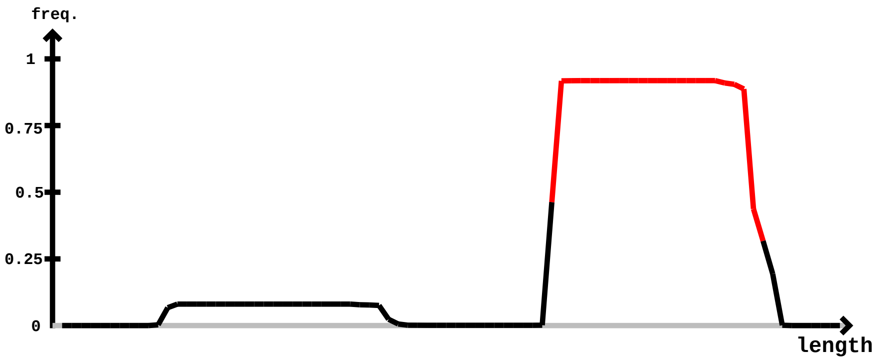

oar-miR-29b

```
5'- cuucuggaagcugguuucacaugguggcuuagauuuuuccaucuuuguaucuagcaccauuugaaaucaguguuuuaggag   -3'    exp
    (((((((((((((((((((.((((((.((.((((..............))))))))))))).)))))))))).)))))))))    reads      mm         sample
    .........gcugguuucacaugguggcuu...................................................    3          0          seq
    .........gcugguuucacaugguggcuuag.................................................    1          0          seq
    .........gcugguuucacaugguggcuuaga................................................    17         0          seq
    .........gcugguuucacaugguggcuuagauuu.............................................    1          0          seq
    ..........cugguuucacaugguggcuu...................................................    12         0          seq
    ..........cugguuucacaugguggcuua..................................................    5          0          seq
    ..........cugguuucacaugguggcuuag.................................................    12         0          seq
    ..........cugguuucacaugguggcuuaga................................................    345        0          seq
    ..........cugguuucacaugguggcuuagau...............................................    98         0          seq
    ..........cugguuucacaugguggcuuagauu..............................................    16         0          seq
    ..........cugguuucacaugguggcuuagauuu.............................................    1          0          seq
    ..........cugguuucacaugguggcuuagauuuu............................................    1          0          seq
    ...........ugguuucacaugguggcuu...................................................    4          0          seq
    ...........ugguuucacaugguggcuua..................................................    1          0          seq
    ...........ugguuucacaugguggcuuaga................................................    43         0          seq
    ...........ugguuucacaugguggcuuagau...............................................    36         0          seq
    ...........ugguuucacaugguggcuuagauu..............................................    18         0          seq
    .................................uuuuuccaucuuuguauU..............................    1          1          seq
    .................................uuuuuccaucuuuguauA..............................    1          1          seq
    .................................uuuuuccaucuuuguauc..............................    6          0          seq
    .................................................ucuagcaccauuugaaaucagu..........    3          0          seq
    ..................................................cuagcaccauuugaaauc.............    31         0          seq
    ..................................................cuagcaccauuugaaauca............    15         0          seq
    ..................................................cuagcaccauuugaaaucag...........    16         0          seq
    ..................................................cuagcaccauuugaaaucGgu..........    24         1          seq
    ..................................................cuagcaccauuugaaaucagu..........    2043       0          seq
    ..................................................cuagcaccauuugaaaucagug.........    709        0          seq
    ..................................................cuagcaccauuugaaaucagugu........    681        0          seq
    ..................................................cuagcaccauuugaaaucaguguu.......    3          0          seq
    ...................................................uagcaccauuugaaauc.............    34         0          seq
    ...................................................uagcaccauuugaaauU.............    2          1          seq
    ...................................................uagcaccauuugaaaucG............    9          1          seq
    ...................................................uaUcaccauuugaaauca............    2          1          seq
    ...................................................uagcaccauuugaaauca............    13         0          seq
    ...................................................uagcaccauuugaaaucGg...........    87         1          seq
```

oar-miR-29b

```
cuucuggaagcugguuucacaugguggcuuagauuuuuccaucuuuguaucuagcaccauuugaaaucaguguuuuaggag
...................................................uagcaccauuugaaaucag...........   31     0     seq
...................................................uagcaccauuugaaaucagu..........   959    0     seq
...................................................uagcaccauuugaaaucGgu..........   400    1     seq
...................................................uagcaccauuugaaaucagug.........   208    0     seq
...................................................uagcaccauuugaaaucagugu........   259    0     seq
...................................................uagcaccauuugaaaucaguguu.......   1465   0     seq
...................................................uagcaccauuugaaaucaguguuu......   9      0     seq
....................................................agcaccauuugaaaucagu..........   1      0     seq
....................................................agcaccauuugaaaucagugu........   1      0     seq
....................................................agcaccauuugaaaucaguguu.......   2      0     seq
.....................................................gcaccauuugaaaucagu..........   1      0     seq
.....................................................gcaccauuugaaaucaguguu.......   1      0     seq
.........................................................cauuugaaaucaguguu.......   1      0     seq
```
